# Chemotherapy-induced Emesis: Experienced Burden in Life, and Significance of Treatment Expectations and Communication in Chemotherapy Care

**DOI:** 10.1177/15347354231217296

**Published:** 2023-12-14

**Authors:** Ylva Widgren, Marit Silén, Ingrid Wåhlin, Magnus Lindberg, Per Fransson, Anna Efverman

**Affiliations:** 1 University of Gävle, Gävle, Sweden; 2 Region Hospital of Sundsvall-Härnösand, County Council of Västernorrland, Sundsvall, Sweden; 3 Region Kalmar, Research Section, Kalmar, Sweden; 4 Umeå University, Umeå, Sweden

**Keywords:** cancer care, communication, expectations, nausea, person-centered care

## Abstract

**Objective:**

Because antiemetics have become more effective and integrative therapies such as acupuncture are used in combination with antiemetics, people receiving chemotherapy for cancer today might expect less emesis than in the past. It is not previously described if and how people receiving modern antiemetics during chemotherapy experience emesis. The objective of this study was to describe experiences regarding emesis among persons undergoing emetogenic chemotherapy, and how it affects their quality of life, daily life and work. A further aim was to describe views on the significance of treatment expectations and communication with healthcare personnel while undergoing chemotherapy for cancer.

**Method:**

Fifteen participants (median age 62 years, n = 1 man and n = 14 women, with breast (n = 13) or colorectal (n = 2) cancer) undergoing adjuvant or neo-adjuvant highly or moderately emetogenic chemotherapy were interviewed individually. The data were then analyzed using inductive thematic analysis.

**Results:**

Three themes described the participants’ experiences: *“Your whole life is affected, or continues as usual,”* covering descriptions of emesis limiting some participants’ everyday lives, while others experienced no emesis at all or had found ways to manage it. Overall, participants described satisfaction with their antiemetic treatment. *“Experiences and expectations more important than information”*, that is, the participants reported wanting all the information they could get about possible adverse effects of treatment, although they believed previous experiences were more important than information in creating expectations about treatment outcomes. The participants reported that being seen as a unique person was of utmost importance: “*Meet me as I am.”* This creates trust in healthcare personnel and a feeling of safety and security in the situation.

**Conclusions:**

These findings underline the importance of person-centered care and support in creating positive treatment expectations. Future research is called for regarding the potential antiemetic effects of positive communication regarding strengthening positive treatment expectations during emetogenic chemotherapy.

## Introduction

People with cancer have traditionally viewed emesis (nausea and vomiting) as one of the most feared side effects of chemotherapy,^1,2^ worsening their quality of life (QoL).^3^ However, less is known about their own views on the link between their expectations regarding nausea^4,5^ and healthcare personnel’s communication^6-10^ in chemotherapy care.

International guidelines on integrative therapy^11^ and antiemetic therapy^12,13^ state that the most effective antiemetic treatment is personalized. The risk of emesis is based on chemotherapy emetogenicity, a person’s previous experiences of chemotherapy-induced emesis, and additional person-related factors that increase this risk. Risks include younger age, female sex, and anxiety.^12^ A widely accepted^14,15^ integrative therapy^16^ for managing chemotherapy-induced emesis that shows promising results is antiemetic acupuncture.^17^ Previous studies have described great non-specific treatment effects of acupuncture.^4,18^ The effects of both conventional and integrative medicine therapies consist of specific and non-specific treatment components. Specific treatment components are, for example, the active substances in a medication^19^ or penetration of the skin and needle stimulation in acupuncture therapy.^4^ Non-specific components include factors surrounding the treatment, for example, treatment expectations. An expectation is a prediction based on reasoning and learning from experience. Conditioning of positive treatment responses through learning from previous experiences and treatment expectation of positive treatment outcomes are suggested to be dominating components beyond the specific components of a treatment that contribute to the total effects on a variety of health outcomes.^19^ Treatment expectations seem to be relevant to emesis-related outcomes during cancer therapy.^4,5^ In a previous study by the first and last author, 68 persons receiving antiemetic acupuncture during chemoradiation therapy rated their belief in acupuncture treatment effects. Only 9 persons of the 29 (31%) who reported great belief in their acupuncture treatment experienced nausea, compared to 17 persons of the 25 (68%) who reported less belief in their acupuncture treatment.^20^ Among 125 women with breast cancer who received chemotherapy, posttreatment vomiting was only predicted by expected severity of vomiting.^21^ These examples indicate that both negative and positive expectations affect the outcomes of antiemetic treatment. Another important non-specific treatment component^19^ seems to be communication between the person undergoing care and the healthcare personnel.^6,7^ The communication from healthcare personnel has been shown to affect treatment expectations.^7-10^ Systematic reviews have demonstrated that positive information about a treatment increased expectations and positively influenced health outcomes such as pain,^10^ recovery,^8^ and psychological conditions.^7^ Research from various care contexts has looked at the clinically significant health effects of these exemplified non-specific treatment components beyond the specific components of the treatment.^7,8,10^ However, there seems to be a need for more studies to gain a more comprehensive understanding of how individuals personally perceive the significance of these non-specific components.^22-24^

There is often a discrepancy in assessing the severity of emesis between the health care personnel and the person with cancer, leading to an underestimation of the negative impact of emesis on the person’s QoL.^25^ Studies using different quantitative measures have shown that people experiencing emesis during chemotherapy perceive worse QoL^3,26^ and less capacity in daily activities^27^ than do people not experiencing nausea. Work ability, that is, capacity to work in relation to workplace demands,^28^ seems to be closely associated with QoL.^29^ Previous research has investigated peoples’ perceptions of various side effects of chemotherapy, such as fatigue and cognitive changes, in relation to work ability.^30,31^ However, there seems to be a lack of knowledge regarding people’s views on their work ability in relation to chemotherapy-induced emesis.

Previous qualitative research has described people’s experiences of chemotherapy-induced emesis.^32-35^ However, the results of these studies might not be applicable in modern cancer care settings. Though it has not previously been described, it seems plausible that, using today’s antiemetics^12,36^ combined with integrative therapies such as acupuncture,^16,17^ emesis is less expected today than in the past.^37^ Integrative cancer care strives to optimize health and empower people to share their experiences and preferences regarding their therapies.^16^ To gain a deeper understanding of people’s care needs during emetogenic chemotherapy used today, descriptions of if and how they experience emesis and its impact on their daily and working life would be welcome. Since chemotherapy-induced emesis might be less expected today, descriptions of how people perceive the significance of treatment expectations and communication with healthcare personnel during antiemetic prophylaxis during chemotherapy would be welcome as well.

### Objective

The study objective was to describe experiences regarding emesis among persons undergoing emetogenic chemotherapy, and how it affects their quality of life, daily life and work. A further aim was to describe views on the significance of treatment expectations and communication with healthcare personnel while undergoing chemotherapy for cancer.

## Method and Materials

### Design

This was a descriptive qualitative interview study,^38^ offering an understanding of experiences during emetogenic chemotherapy. The study was approved by the Regional Ethical Review Board in Umeå (2016/362-31).

### Participants

The participants in this study were recruited from a clinical trial conducted during their chemotherapy period (clinicaltrials.gov #NCT03232541) at an oncology clinic in northern Sweden. In that trial, participants undergoing adjuvant or neo-adjuvant emetogenic chemotherapy for cancer received either penetrating or non-penetrating acupuncture in addition to antiemetics, or just standard care with antiemetics alone. They also unknowingly received neutral or positive communication from the treating therapist regarding the effect of the antiemetic acupuncture or the antiemetics. The clinical trial was conducted during one of the participant’s chemotherapy sessions. The inclusion criteria: Diagnosed with breast, colorectal, bladder, or testicle cancer, age ≥18 years, currently receiving or having completed curative adjuvant or neo-adjuvant intravenous chemotherapy of medium or high emetogenicity,^12^ participated in the clinical trial during their chemotherapy period, and physical, mental and linguistic capacity to give informed consent and participate in study procedure, that is, understand Swedish. Exclusion criteria: More than 1 year since participation in the randomized clinical trial.

The study coordinator (the first author) sent a letter with information about the interviews to 28 strategically selected participants in the clinical trial, the aim being to achieve variation in sex, age, cancer types, and antiemetic treatment type (acupuncture using penetrating or non-penetrating needles in addition to antiemetics or antiemetics alone). The study coordinator called the selected participants a few days after the study information was sent to provide verbal study information and ask whether they were interested in being interviewed. Of the 28 informed participants, 6 declined the invitation right away, due to health conditions or for practical reasons. Three were willing to participate “but not now,” and 2 did not answer the phone. The remaining 17 provided their written informed consent. Two of them later declined to take part in the interview for personal reasons. Based on the criteria regarding information power,^39^ we considered 15 participants to be a reasonable number for this study. The characteristics of the participants are presented in Table 1.

**Table 1.** Characteristics of the Participants.

	Variable	Participants, n = 15
Sociodemographic characteristics	*Age in years, md (range)*	62 (42-74)
	*Sex, n (%)*	
	Men	1 (7)
	Women	14 (93)
	*Civil status, n (%)*	
	Married/living with partner	10 (67)
	Living alone with partner	1 (6)
	Living alone without partner	4 (27)
	*Education level, n (%)*	
	Elementary school	3 (20)
	High school/ occupational education^a^	7 (47)
	University	5 (33)
	*Work situation, n (%)*	
	Employed, on sick leave	7 (47)
	Employed, not on sick leave	1 (6)
	Retired, on disability pension	7 (47)
Clinical characteristics	*Cancer type, n (%)*	
	Breast	13 (87)
	Colorectal	2 (13)
	*Had completed chemotherapy at the time of the interview, n (%)*	
	No, still undergoing chemotherapy	1 (7)
	Yes	14 (93)
	Weeks between end of chemotherapy and interview, md (range)	11 (1-44)
	*Received antiemetic acupuncture in the clinical trial, as a complement to standard care, n (%)*	
	No	4 (27)
	Yes^b^	11 (73)
	*Quality of life (mm EuroQoL-VAS)*^c^ *day before chemotherapy*,^d^ *md (range)*	n = 14 74 (40-100)

Abbreviation: Md, median.

a Not on university level.

b Given with penetrating or non-penetrating needles.

c VAS: Visual Analog Scale, graded 0, worst QoL imaginable, to 100, best QoL imaginable.^40^

d The chemotherapy session when they participated in the clinical trial.

### Data Collection

#### Demographics, clinical data and quality of life

The study coordinator collected descriptive data on demographics and QoL the day before participants participated in the clinical trial. A study nurse at the oncology clinic collected clinical data on cancer type from medical records (Table 1).

#### Interview data

A physiotherapist (last author, AE, experience from clinical settings [20 years], and from interviewing [3 interview studies]), involved neither in the cancer therapy nor in the antiemetic treatment, individually interviewed^38^ the participants once (at least 10 days after receiving chemotherapy). The interviews were held at a place chosen by the participant, mostly at the hospital but sometimes in the participant’s home. A semi-structured interview guide (Appendix) was used containing 6 open-ended questions. Follow-up questions were posed if the participant needed support in telling his/her story. The questions focused on experiences of emesis during chemotherapy. Further, questions concerning participants’ expectations concerning emesis and antiemetic treatment were posed. Finally, participants were asked questions about communication: how they experienced communication from the healthcare personnel during chemotherapy; how they think healthcare personnel should communicate; how they viewed the role of communication in treatment effects and shaping expectations. The interviews were audio-recorded in their entirety (ranging in duration 22-52 minutes; md 32 minutes) and then transcribed verbatim by the first author (YW). Prior to the study, the interview guide was tested during 2 pilot interviews with patients undergoing chemotherapy. Interview question number one was changed after the pilot interviews, from “have you had any problems with nausea, and if so what kind?” to “how has it been for you regarding nausea, if you had any?” The goal of this change was to avoid indicating that nausea had to be a problem. The 2 pilot interviews were not included in the study.

### Data Analysis

The researchers mainly involved in the analysis were the first (YW), second (MS) and third (IW) authors. YW has worked as a physiotherapist in cancer rehabilitation for many years. MS and IW are nurses with different clinical backgrounds and long experience in qualitative research. YW, new to qualitative research, performed an inductive thematic analysis using a reflexive approach with semantic focus, meaning that the analyst was not looking for anything beyond what a participant said.^41^ Because our focus of inquiry was on participants’ subjective experiences of emesis, treatment expectations and communication during chemotherapy, this approach was well suited to our research aim.

In reflexive thematic analysis, researchers, especially those new to the method, are encouraged to review and reflect with their co-researchers at all stages of the research process.^41^ Thus, after becoming familiar with the interview data, YW and MS read half of the interviews each more thoroughly and selected data from each interview that were relevant to the study objective. The selected data was then coded, and YW and MS provided feedback on each other’s coding (see Table 2 for examples of data and codes). The next step was to generate initial themes. This was done by YW, with constructive feedback from MS and IW. The themes were then discussed with all the other authors. The initial themes were thereafter refined into more developed themes by moving back and forth between the codes and data in a reflexive manner. The themes were then defined additionally and, after feedback from the other authors, finally named. The last step in the analysis was to use the analyzed data to write an analytic story. During the entire writing process, the analyzers actively discussed the themes and did not decide on their final form until the manuscript was finished. The aim of having the more experienced co-researchers review and reflect on the analysis throughout the entire process was to test and question the analytic ideas and assess whether they stood up to critical questioning and scrutiny, thus enhancing the quality of the findings.^41^

**Table 2.** Examples of Data and Codes.

Data	Codes
People are individuals and they have a lot, it depends on what you’ve been through and how easily you deal with nausea, for me well, yeah, I didn’t have so much nausea, I was fine with the medicines the health-care personnel sent home with me (Participant 9)	Didn’t have so much nausea. Was fine with the medicines [antiemetics] she was sent home with.
Well, I don’t like taking pills, generally speaking, but I’ve realized I have to, that’s the way it is, so. And they do help. (Participant 12)	Took antiemetics because she had to. The pills helped reduce nausea.
I know I bought cauliflower, carrots and other things for my first treatment, and if I ate a piece of cauliflower or carrot it reduced it [nausea] (Participant 7)	Ate vegetables to reduce nausea, and it helped.

Examples of codes related to data are described.

## Results

**Figure 1.**
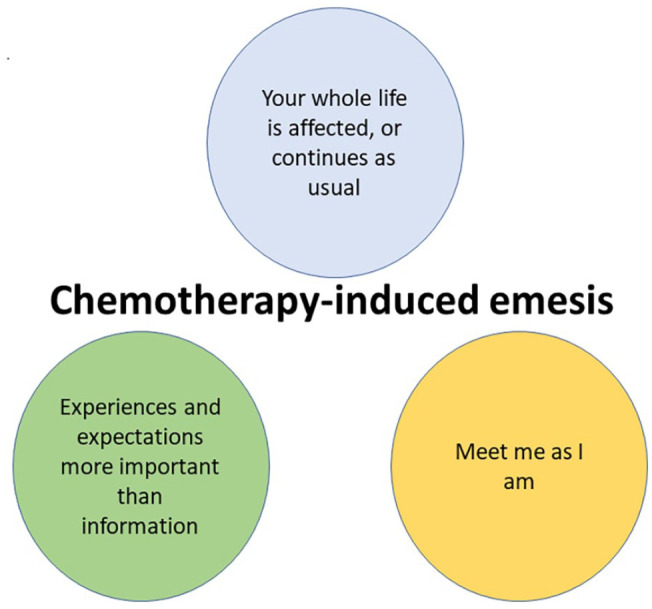
The 3 themes describing experiences among persons undergoing emetogenic chemotherapy.

The thematic analysis resulted in 3 themes, illustrated in Figure 1: “Your whole life is affected, or continues as usual,” “Experiences and expectations more important than information,” and “Meet me as I am” (Figure 1).

### Theme 1: Your Whole Life is Affected, or Continues as Usual

Some participants did not experience much nausea at all, which allowed them to continue living as usual, while others had to adapt their entire lives to their nausea. This contradiction appeared throughout the whole data set.

Those who experienced nausea mainly did so during the first and second week after chemotherapy. The third week post-treatment was often described as the “good” week. Then it was time for the next chemotherapy session and for everything to start over again.

Some participants reported not being able to work, either because of the nausea itself or the tiredness that came with it. As one of the participants described it:

“. . . if you’re feeling sick then being at work isn’t a good idea, it’s like really not (laughs). . . And then you don’t have the energy to be up all day either. . . then you’d have to have a bed at the office (laughs). . .” (Participant 10)

Whether and to what extent it was possible to work depended, among other things, on the type of work and the possibility to have flexible workdays. The participants who could still work were mainly those who could adapt their workdays in different ways, for example, resting when needed and compensating by working when they felt better.

Outside work, nausea limited some participants’ everyday life and everything that belonged to it, like making food, taking a shower, doing the laundry or cleaning up a bit. Not being able to perform such everyday tasks was experienced as very frustrating. The nausea also limited their ability to meet with friends and colleagues. The participants handled the situation in different ways. Some reacted apathetically and just sat at home doing almost nothing. Others got help with, for example, cooking from friends and family members and became masters of identifying what and when to eat. Meetings with friends and other social activities were scheduled during the weeks when they felt better:

“. . . I made calculations, so I could say, okay, we’ll come and visit, or we, we’ll do this and that, we’ll go here or there. . . then I knew it was the third week that worked. . .” (Participant 2)

There were also participants who experienced no nausea at all and, thus, never needed to take the prescribed extra antiemetics:

“. . . Well, I haven’t felt nauseous at all actually. . . I’ve been fine, I got some huge pills at first that we tested . . . and I didn’t feel anything. . . so we thought let’s try without them. . . and it went really well . . .” (Participant 1)

Overall, the participants expressed great confidence in the antiemetics they were given, both the medication and the antiemetic acupuncture, and felt that they had helped:

“. . . I think the pills really helped. . . Well, they helped me anyway. . .” (Participant 6)

### Theme 2: Experiences and Expectations More Important Than Information

Participants reflected on how treatment expectations are created. The reflections were not limited to merely expectations concerning the emesis treatment, but the entire chemotherapy and its potential side effects. Previous experiences, compared to information, were reported to be more important for creating expectations about whether or not the treatment would be effective. These could be their own or others’ experiences, for example, those of relatives who had undergone chemotherapy or people they had met when working in healthcare. The participants’ own experiences from previous chemotherapy sessions sometimes affected expectations so greatly that the person felt nauseous when merely thinking about the next treatment session:

“. . .. Well, when I think about getting the treatment next week, I get sick automatically, I feel nauseated. . .” (Participant 11)

Positive experiences also affected expectations, for example, positive experiences of acupuncture treatment earlier in life created positive expectations, in this case regarding the antiemetic acupuncture treatment:

“. . . I’ve had acupuncture for headaches. . . and that worked really well. . . so my expectations were actually very great. . .” (Participant 1)

The participants reported that their experiences and feelings about treatment played a greater role than the healthcare personnel’s information and positive talk about the treatment. They said that if patients enter the treatment with a negative attitude, it is hard for healthcare personnel to change that with positive talk. They also reported that feelings of, for example, nausea do not change, regardless of how many people tell you that the nausea should be better today. What matters the most is how you experience your own symptoms. That is your truth.

Despite this, most participants nonetheless reported wanting all the information they could get about their chemotherapy and other medications. As one of the participants described it:

“. . . it’s better if they inform you that this may be a problem, and then it isn’t . . . that’s like better than the reverse. . .” (Participant 15)

### Theme 3: Meet me as I am

Participants talked about the importance of being seen as a unique person by healthcare personnel, reporting that this greatly influenced their experiences and expectations. This could involve anything from healthcare personnel remembering your name to taking time to sit with you during treatment. The healthcare personnel’s ability to care about and see individual patients was considered just as important a quality as having all kinds of knowledge.

“. . . I think it’s the most important thing, along with knowledge/. . ./ that you know someone cares, kind of like they see you. . .” (Participant 13)

The participants also described being involved in treatment decisions as important. They wanted to feel that both the treatment information and the treatment itself were individualized. This made them feel listened to and respected.

“. . . it’s pretty individual, you know, how I feel, and then they adapt it to me/. . ./it feels like there are lots of conversations about, well, how I am doing. . . and then the discussion revolves around that. . .” (Participant 15)

Moreover, if participants were able to meet and talk with the healthcare personnel pre-treatment, trust was created. This personal connection made the participants feel secure and safe going into the upcoming treatment situation:

“. . . when I came to [the hospital for the operation]/. . ./I got to meet with all of them [all personnel] the day before. . . so when I came in for the operation. . . well, I felt so safe/. . ./ because when you’ve talked to the person and gone through everything, he knew everything, like. . . about me. . .” (Participant 8)

## Discussion

The present study found that some participants receiving emetogenic chemotherapy felt like nausea was limiting their everyday lives, while others experienced no nausea at all or had found ways to manage it. In the results it was evident that participants reported wanting all the information they could get about possible adverse effects of the treatment, even though they believed previous experience was more important than information for creating expectations about the treatment. The participants reported that to be seen as a unique person was of the utmost importance. That created trust in the healthcare personnel and a feeling of safety and security in the treatment situation.

The central finding in our first theme is the contradictive experiences regarding emesis among the study participants. Contradiction within a theme is not typically recommended in reflexive thematic analysis, but contradiction *can* appear when the contradiction is what the theme is *about*.^41^ In our study, the participants’ daily lives were either completely affected or not affected at all by emesis. We thus considered the dichotomization within the theme to be central.

Variation in experienced nausea intensity during chemotherapy has previously been reported,^33,34^ but our study differed in that some participants seemed to have experienced no nausea at all, and only one participant reported vomiting and that was only on one occasion. This could plausibly be explained by today’s antiemetic treatment in chemotherapy care being more effective and personalized today than previously, when the other studies were conducted.^13^ To achieve optimal emetic prophylaxis, the antiemetic guidelines must be followed. That is not always the case, as shown in a retrospective study where a review of 299 patient records and prescriptions was conducted. Only 61% and 11% of patients received correct prophylaxis for acute and delayed chemotherapy-induced emesis, respectively.^42^ The participants in our study were all from the same clinic and given that some of them did not experience much nausea at all, one might speculate that the antiemetic guidelines were probably adhered to by their physicians. Further, 11 of the 15 interviewed participants received antiemetic acupuncture in addition to their antiemetics, which has previously been shown to be associated with satisfied patients.^17^

The level of daily or work activities of some participants was affected. The ability to work varied among participants and seemed to depend on the type of work they had. Participants who had flexible office hours and could work from home seemed to have an advantage over those who had fixed hours at their workplace. With flexible workdays, it was possible to rest when needed and then compensate at other times of the day, which was not possible for those working, for example, at a school or daycare center. This perceived difference in work ability related to type of work seems to be a new finding regarding ongoing chemotherapy, but was in line with previous research regarding long-time work, where adjustments in work, such as flexibility regarding work hours, has been seen to strongly predict positive long-term work outcomes in persons with cancer.^43^ However, this was not seen in women with cognitive side effects following chemotherapy, where cognitive functioning affected all types of work.^31^ Our promising finding indicates that it seems possible for many people with chemotherapy-induced emesis to work during their cancer treatment if their work is flexible regarding both time and location.

The nauseated participants reported not being able to perform everyday tasks or meet with friends like they used to. This limitation of social life seems to be a common problem among people with chemotherapy-induced emesis. Previously interviewed women with gynecological cancer reported that nausea hindered participation in social activities, causing them to consider whether continuing treatment was worth it.^44^ The participants in our study tackled this in different ways, either using problem-focused managing strategies like scheduling activities on days they knew they would probably feel better, or more emotion-focused strategies like handling the situation with humor. Similarly, previously interviewed women (n = 9) experiencing chemotherapy-induced emesis reported using both problem- and emotion-focused strategies for managing their nausea. Problem-focused strategies could involve going shopping with friends or taking care of the household, while emotion-focused strategies could be positive thinking or visualization.^32^ The attribute of maintaining hope and positivity has been discussed as both a need and an important strategy for managing the cancer disease.^23,24,45^ People with cancer in a previous qualitative study (n = 25) described being positive as a strategy associated with better outcomes, whereas being negative was counter-productive concerning treatment outcomes.^45^

In our results it was evident that participants in our study reported wanting information about their treatment and possible adverse effects. This is in line with findings in a recent systematic review, where persons newly diagnosed with cancer indicated that receiving information about the cancer itself and treatment options was very important. They considered information about side effects to be important but found provision of such information to be insufficient,^46^ in contrast to our interviewed participants who felt they were well-informed. The novel finding in our study is that regardless of how much information the participants want and get, they still believe that previous experience is the most important factor for creating expectations about the upcoming treatment.

Learning from previous situations and thus forming expectations is a key component of general theories on mechanisms for shaping new experiences.^19^ Interestingly, the present findings confirm this key component in this clinical context. There were descriptions that merely thinking about the upcoming chemotherapy session, which was associated with expected emesis, induced nausea several days before the actual treatment day. Such conditioned, anticipatory nausea^2^ demonstrates the great significance of expectations^4,5^ and the importance of effective antiemetic treatments in avoiding severe breakthrough emesis, which can lead to development of anticipatory nausea. The affected person develops a strong conditioned response, which has both psychologic and physiologic components.^2^

There were extraordinary expressions of only wanting hopeful information and positive examples from the healthcare personnel. Similarly, previously interviewed persons undergoing cancer therapy expressed a need for healthcare personnel to encourage hope during patient interactions.^45^ However, most of our interviewed participants wanted honest and fact-based information about their diagnosis and its treatment.

The participants expressed a desire for healthcare personnel to see them for who they are and to adapt information and treatment to the unique person in front of them. Some people want and need a great deal of encouragement and positive talk about the treatment, and others do not. The challenge for healthcare personnel is to identify each person’s desires and needs. This is in line with the ideas of integrative cancer care^16^ and person-centered care in general, where individuals’ values and preferences guide all aspects of their health care, supporting their realistic health and life goals.^47^ When providing medical information, it is important to take the individual person’s managing styles into account, as providing information to a person who prefers to avoid medical information will only be counter-productive.^48^

Reviewing the methodology used in our study, our intention in including the participants was to achieve variation in sex, age, cancer types, and antiemetic treatment type (penetrating or non-penetrating acupuncture in addition to antiemetic medication, or antiemetics alone). We did achieve variation in age and antiemetic treatment type, but not in sex and cancer type. This can be explained by the fact that the participants were selected from a clinical trial, in which the majority of participants had breast cancer. Of the 28 strategically selected participants, 8 were men. The men had a higher tendency to decline participation than the 20 women, which was unfortunate but not surprising, as women have been shown to be more willing to participate in health research than men.^49^ Hence, in the end we had 14 women and 1 man for the interviews. Given that emesis is more common in women than in men^12^ and that the study concerned experiences of chemotherapy-induced emesis, the predominance of female participants can be seen as beneficial to the study. However, if a few more men had been included, preferably younger men, the data might have been more varied.

Regarding data collection, it was obvious that, during the interviews, many of the participants had difficulties remembering the conversation with the therapist providing their antiemetic treatment. This may not be surprising, because 11 weeks (median value) had passed since they completed their chemotherapy. The meager recollections of the therapist’s communication, neutral or positive, affect the credibility of the study, that is, how well the data address the intended focus in this respect. Therefore, it might have been advantageous to interview the participants closer in time to their meeting with the therapist. On the other hand, the participants had more time to reflect on their experiences as time passed since their chemotherapy.

It is not a typical part of reflexive thematic analysis to try to eliminate subjectivity as a source of bias. Instead, subjectivity is embraced as a resource.^50^ None of the authors involved had a personal history of cancer or chemotherapy-induced emesis, perhaps making them more open to and curious about the participants’ experiences, but also perhaps limiting their ability to understand them. Given that all analyzers are women with a healthcare background, it was continuously important to consider why we interpreted the data in the way we did. We encouraged each other throughout the analytic process, giving input on each other’s interpretation. We applied the “20 best practice recommendations” (except for a combined result- and discussion chapter) for good quality standard in conducting and reporting a thematic analysis.^50^ Other quality standards contain problematic assessment tools for reflexive thematic analysis, given their strong emphasis on reaching analytic consensus between authors. In reflexive thematic analysis, the emphasis is on authors helping to enhance understanding rather than on reaching consensus.^50^

Considering the important findings showing that some participants experienced no emesis at all and the perceived significance of treatment expectations and communication with healthcare personnel, we propose that people beginning chemotherapy should receive that promising information. They would then plausibly expect less, and hypothetically also experience less, emesis. Future research is called for to investigate the potential antiemetic effects of positive communication that strengthens positive treatment expectations during emetogenic chemotherapy.

## Appendix

**Appendix.** The Semi-Structured Interview Guide.

Interview questions	Research areas
1. Can you tell me about your experiences during your chemotherapy treatment; how has it been for you regarding nausea, if you had any?	Emesis
2. Tell me your expectations concerning nausea and the antiemetic treatment with acupuncture and/or medications that you received?	Expectations
3. During your antiemetic acupuncture/medical treatment the time you participated in the clinical trial A) How did you experience the communication with the therapist? B) What importance does communication have during treatments? Then the interviewer revealed to the participant in what way, positively or neutrally, the therapist had been communicating with him or her during the antiemetic treatment in the RCT, and asked: 4. What are your thoughts regarding the fact that the therapist communicated in that way with you? 5. Do you think that the way that health care personnel communicate about expected treatment effects can affect treatment results and if so, in what way? 6. How would you like health care personnel to communicate during treatment?	Communication

The interview questions divided into the 3 research areas (1) emesis, (2) expectations, and (3) communication. The questions were not always asked in this specific order.
